# Impact of alcohol abstinence on survival after hepatic resection for hepatocellular carcinoma in patients with alcohol-related liver disease

**DOI:** 10.1016/j.amsu.2021.102644

**Published:** 2021-07-29

**Authors:** Daisuke Shirai, Hiroji Shinkawa, Shigekazu Takemura, Shogo Tanaka, Ryosuke Amano, Kenjiro Kimura, Masahiko Kinoshita, Norifumi Kawada, Shoji Kubo

**Affiliations:** a Department of Hepato-Biliary-Pancreatic Surgery, Osaka City University Graduate School of Medicine, 1-4-3 Asahimachi, Abeno-ku, Osaka, 545-8585, Japan; b Department of Hepatology, Osaka City University Graduate School of Medicine, 1-4-3 Asahimachi, Abeno-ku, Osaka, 545-8585, Japan

**Keywords:** Alcohol abstinence, Alcohol-related liver disease, Hepatocellular carcinoma, APRI, aspartate aminotransferase-to-platelet ratio index, ALD, alcohol-related liver disease, ALT, alanine aminotransferase, AST, aspartate aminotransferase, BMI, body mass index, FIB-4, fibrosis index based on four factors, GGT, gamma-glutamyl transpeptidase, HBV, hepatitis B virus, HCC, hepatocellular carcinoma, HCV, hepatitis C virus, OS, overall survival, RFS, recurrence-free survival, TACE, transcatheter arterial chemoembolization

## Abstract

**Background:**

This study aimed to evaluate the prognostic impact of alcohol abstinence on survival after hepatic resection for hepatocellular carcinoma (HCC) in patients with alcohol-related liver disease (ALD).

**Patients and methods:**

In total, 92 patients with ALD-HCC who underwent initial and curative hepatic resection were identified, including 56 and 36 patients with and without alcohol abstinence, respectively.

**Results:**

The 3-, 5-, and 7-year recurrence-free survival (RFS) were 46%, 43%, and 37% in the abstinence group, and 61%, 36%, and 36% in the non-abstinence group, respectively (p = 0.71). The 3-, 5-, and 7-year overall survival (OS) were 91%, 76%, and 66% in the abstinence group, and 87%, 57%, and 44% in the non-abstinence group, respectively (p = 0.023). Multivariate analysis revealed that non-abstinence was an independent prognostic factor for OS (P = 0.026). The incidence rate of liver-related death including HCC-specific death, liver failure, and renal failure in cirrhosis (hepatorenal syndrome) between the non-abstinence and abstinence groups were 41.7% vs. 19.6% (p = 0.032). Worsening of the Child–Pugh grade at intrahepatic recurrence was more frequently observed in the non-abstinence (33.3%) than that in the abstinence group (6.5%) (p = 0.039).

**Conclusions:**

Alcohol abstinence might improve the long-term survival of patients with ALD-HCC undergoing hepatic resection.

## Highlights

• Non-abstinence after surgery was an independent prognostic factor for overall survival with a 2.2-fold increased risk.
• Worsening of Child–Pugh grade at intrahepatic recurrence was less frequently observed in the abstinent patients.
• Hepatic resection tended to be more frequently performed for the treatment of HCC recurrence in the abstinent patients.
• The incidence rate of liver-related death was significantly lower in the abstinent patients.

## 1 Introduction

Hepatocellular carcinoma (HCC) is one of the most common cancers worldwide [1,2]. Its occurrence is closely related to the presence of chronic liver disease, mostly as a result of hepatitis B or C virus (HBV or HCV) infection. However, the proportion of patients with HCC who are negative for hepatitis B surface antigen (HBs Ag) and anti-HCV antibody has recently increased [[3], [4], [5]]. Without HBV or HCV infection, lifestyles such as dietary habit, smoking, physical activity, and alcohol consumption have been reported to increase the risk of HCC development [[6], [7], [8]]. Alcohol abuse is one of the causes of HCC, reported to be responsible for approximately 30%–45% increase of HCC incidence in Western countries [5,9,10].

Alcohol-related liver disease (ALD) is manifested by alcohol-induced liver inflammation leading to fibrosis and carcinogenesis progression [11,12]. The most effective treatment for patients with ALD is the achievement and maintenance of alcohol abstinence due to its limited effective medical treatment [13]. Previous reports indicated that alcohol abstinence would improve the long-term prognosis in patients with ALD including alcoholic steatohepatitis and alcoholic cirrhosis [14,15]. In patients undergoing liver transplantation for ALD, alcohol recidivism is reported to impair long-term survival after liver transplantation [16,17]. However, in patients undergoing hepatic resection for HCC associated with ALD (ALD-HCC), to the best of our knowledge, no report has evaluated the impact of alcohol abstinence on the long-term survival postoperatively.

Therefore, this study aimed to evaluate the prognostic impact of alcohol abstinence on survival outcome after hepatic resection for ALD-HCC.

## 2 Patients and methods

### 2.1 Patients

A total of 92 patients with ALD-HCC who underwent initial and curative hepatic resection at the Osaka City University Hospital from January 1994 to December 2018 were identified. ALD was diagnosed according to chronic heavy alcohol use (alcohol consumption of >60 g/day for >5 years) with the following histological features: steatosis, hepatocellular injury with ballooning, lobular inflammation, fibrosis or cirrhosis [18], and exclusion of other causes of liver disease, such as HCV or HBV infection, autoimmune hepatitis, primary biliary cirrhosis, and Budd–Chiari syndrome [19]. Curative hepatic resection was defined as complete resection of a recognizable tumor and the histological absence of tumor cells along the parenchymal transection line. This study was approved by the guidelines of our institutional ethics committee (no. 3815). This study was conducted in accordance with the Declaration of Helsinki and reported in line with the Strengthening the Reporting of Cohort Studies in Surgery 2019 guidelines [20].

### 2.2 Alcohol abstinence

All patients were advised to abstain from alcohol. Alcohol intake was evaluated through patients’ self-report and family member interviews by primary doctors. Alcohol abstinence was defined as drinking occasionally, a monthly alcohol intake of less than once with <20 g/day of alcohol intake [14]. Of 92 enrolled patients, 56 achieved persistent abstinence from alcohol postoperatively without relapse (abstinence group) and 36 patients did not abstain from alcohol postoperatively (non-abstinence group).

### 2.3 Patient follow-up

All patients were followed up 1 month postoperatively and 3 months thereafter. Follow-up evaluations were as follows: physical examination, liver function tests, HCC-specific tumor marker, chest radiographs to examine for pulmonary metastases, and ultrasonography, dynamic computed tomography, or magnetic resonance imaging to examine for recurrence in the remnant liver or other abdominal organs. For patients with recurrence, appropriate therapeutic treatment was adopted such as re-hepatic resection, radiofrequency ablation, or percutaneous ethanol injection therapy defined as curative treatment, and transcatheter arterial chemoembolization or other treatment alternatives, as non-curative treatment [21]. Overall death was categorized into liver-related death and other causes of death. Liver-related death included HCC-specific, liver failure, and renal failure in cirrhosis (hepatorenal syndrome) [22]. The median follow-up period was 45.5 (interquartile range, 23.4–76.3) months in the overall cohort. Of the 92 patients, 29 (31.5%) dropped out during the follow-up; the median follow-up period was 41.8 (interquartile range, 17–65.4) months for the patients who dropped out.

Gamma-glutamyl transpeptidase (GGT) and liver fibrosis indices including aspartate aminotransferase (AST) to platelet ratio index (APRI) and fibrosis index based on four factors (FIB-4) index were also evaluated for available patients postoperatively at our outpatient. Liver fibrosis indices were calculated using the following formulas: APRI = [(AST/upper limit of normal)/platelet count (10^9^/L)] × 100; FIB-4 = age (years) × AST (U/L)/[platelet count (10^9^/L) × (alanine aminotransferase [ALT] [U/L])^1/2^].

### 2.4 Histology

The guidelines of the Liver Cancer Study Group of Japan [23] were used to evaluate histological tumor classifications and degree of the background liver. The grade (active hepatitis severity) and stage (degree of hepatic fibrosis) of non-cancerous hepatic tissue were determined by scoring based on the histologic activity index [24,25].

### 2.5 Statistical analysis

Categorical variables were compared using Fisher's exact test. Continuous data were compared using the Mann–Whitney *U* test. Rates of recurrence-free survival (RFS) and overall survival (OS) were evaluated using the Kaplan–Meier method. Differences between curves were evaluated using the log-rank test. RFS and OS were evaluated by univariate and multivariate analyses using the Cox proportional hazards model. Variables potentially associated with recurrence were selected based on previous study results or on our own clinical experience, including age (≤65 or >65 years), gender, body mass index (BMI) (≤25 or >25 kg/m^2^), serum GGT (<65 or ≥65 U/L), ALT activity (≤30 or >30 U/L), albumin concentration (>3.5 or ≤3.5 g/dL), prothrombin time activity percentage (>70 or ≤70%), total bilirubin level (≤1.0 or >1.0 mg/dL), serum alpha-fetoprotein (≤20 or >20 ng/mL), Child–Pugh grade, tumor size (>3.0 or ≤3.0 cm), degree of tumor differentiation (poorly or well/moderate), microscopic portal invasion, multiple tumors, liver cirrhosis, grading score (0–2 or 3–4), and major hepatic resection (≥2 section). Variables with a *P*-value of <0.1 in univariate analysis were entered into the multivariate analysis. The threshold for statistical significance was set at P < 0.05. Statistical analyses were performed using the SPSS software v21.0 (IBM Corp, Armonk, NY) and EZR (Saitama Medical Center, Jichi Medical University, Saitama, Japan).

## 3 Results

### 3.1 Patient characteristics

Clinicopathological characteristics of 92 patients are shown in Table 1. The mean age was 68 (range, 41–82) years, and patients comprised of 89 men and 3 women. The number of patients with BMI of >25 kg/m^2^ were significantly higher in the non-abstinence than that in the abstinence group (p = 0.016). Proportions of >3-cm tumor size and major hepatic resection were significantly higher in the abstinence group than that in the non-abstinence group (p = 0.028 and p < 0.001, respectively).

**Table 1:** Patient characteristics.

Variable	Non-abstinence group (n = 36) (%)	Abstinence group (n = 56) (%)	*P*
Age >65 years	23 (63.9)	37 (66.1)	0.83
Gender: male	34 (94.4)	55 (98.2)	0.56
BMI >25 kg/m^2^	20 (55.6)	16 (28.6)	0.016
GGT ≥65 U/l	21 (58.3)	33 (58.9)	>0.99
ALT >30 U/l	16 (44.4)	27 (48.2)	0.83
Albumin ≤3.5 g/dL	3 (8.3)	11 (19.6)	0.23
PT activity ≤70%	3 (8.3)	1 (1.8)	0.30
Total bilirubin >1.0 mg/dL	6 (16.7)	10 (17.9)	>0.99
α-fetoprotein >20 ng/mL	6 (16.7)	20 (35.7)	0.059
Child-Pugh grade: A	35 (97.2)	55 (98.2)	>0.99
Tumor size >3 cm	17 (47.2)	40 (71.4)	0.028
Multiple tumor	7 (19.4)	15 (26.8)	0.46
Tumor differentiation (poor)^#^	7 (19.4)	9 (16.1)	0.78
Microscopic portal invasion	6 (16.7)	17 (30.4)	0.22
Liver cirrhosis	10 (27.8)	12 (21.4)	0.62
Grading score ≥3	3 (8.3)	4 (7.1)	>0.99
Major hepatic resection	1 (2.8)	20 (35.7)	<0.001

BMI, body mass index; GGT, Gamma-glutamyl transpeptidase; ALT, alanine aminotransferase; PT, prothrombin time; ^#^tumor differentiation: poor, poorly differentiated.

### 3.2 Survival outcome

**Fig. 1:**
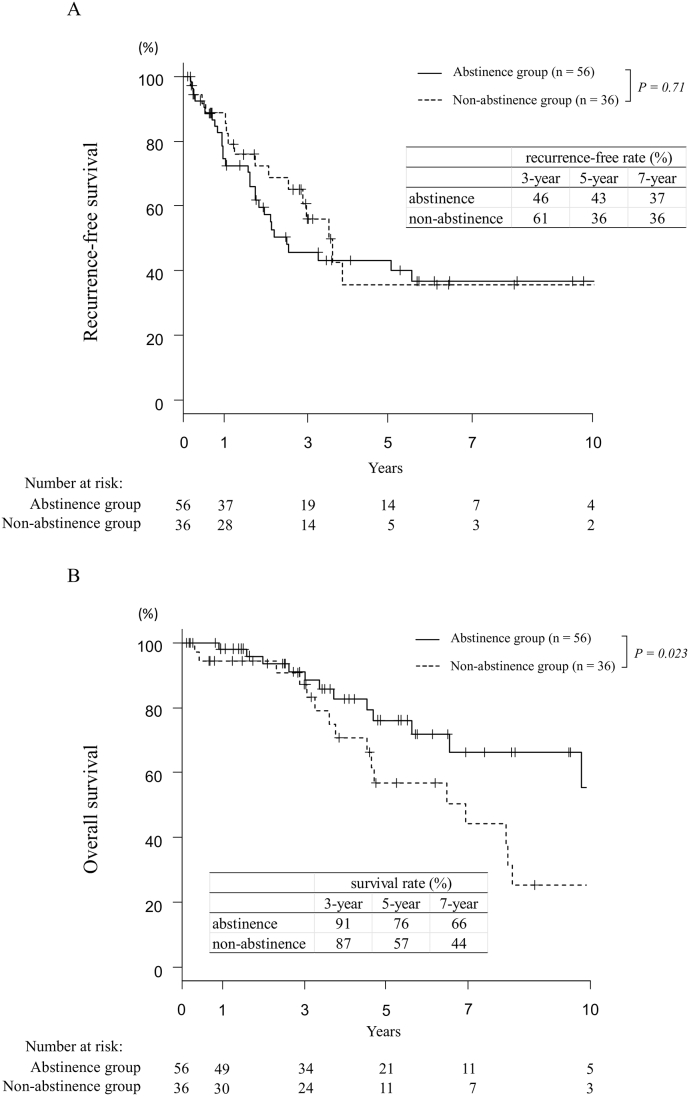
Survival outcomes in abstinence patients and drinking after hepatic resection A: Recurrence-free survival B: Overall survival

Fig. 1A and B shows the RFS and OS after hepatic resection. The 3-,5-, and 7-year RFS were 46%, 43%, and 37% in the abstinence group, and 61%, 36%, and 36% in the non-abstinence group, respectively (p = 0.71). The 3-,5-, and 7-year OS were 91%, 76%, and 66% in the abstinence group, and 87%, 57%, and 44% in the non-abstinence group, respectively (p = 0.023).

Univariate analysis showed that male gender (p = 0.078), liver cirrhosis (p = 0.069), and non-abstinence (p = 0.023) were associated with a lower OS rate (Table 2). Multivariate analysis revealed that non-abstinence was an independent prognostic factor for OS (hazard ratio, 2.24; 95% confidence interval [CI] 1.1–4.54; P = 0.026) (Table 3).

**Table 2:** The overall survival postoperatively.

Variables	Number	MST	Survival rate (years)			*P*
		(days)	3	5	7	
Age (years)						
≤65	32	3567	94	75	67	0.11
>65	60	2534	85	65	46	
Gender						
Female	3	1657	67	0	0	0.078
Male	89	2952	89	71	59	
BMI kg/m^2^						
≤25	56	3567	92	66	58	0.58
>25	36	2894	86	72	55	
GGT (U/l)						
<65	40	NA	85	73	55	0.75
≥65	52	2916	98	75	62	
ALT (U/l)						
≤30	49	2372	88	61	47	0.23
>30	43	3567	90	73	64	
Albumin (g/dL)						
>3.5	78	2916	90	69	57	0.52
≤3.5	14	3567	84	64	64	
PT activity (%)						
>70	88	2952	89	70	59	0.76
≤70	4	1657	75	50	50	
Total bilirubin (mg/dL)						
≤1.0	76	3567	92	76	61	0.18
>1.0	16	1709	80	42	42	
α-fetoprotein (ng/ml)						
≤20	66	2952	89	72	61	0.99
>20	26	3567	91	59	50	
Child–Pugh grade						
A	90	2952	89	69	58	0.14
B, C	2	1317	NA	NA	NA	
Tumor size (cm)						
>3.0	57	2952	90	76	60	0.31
≤3.0	35	2054	89	54	48	
Differentiation degree^#^						
Well, mod	76	2916	90	70	57	0.87
Poor	16	3567	88	62	62	
Microscopic portal invasion						
Presence	23	NA	90	51	51	0.69
Absence	69	2952	89	73	61	
Tumor number						
Single	70	2894	88	64	51	0.14
Multiple	22	3712	94	80	71	
Liver cirrhosis						
Presence	22	1721	88	49	37	0.069
Absence	70	3668	90	74	59	
Grading score						
0–2	85	2952	90	67	59	0.95
3–4	7	2394	83	67	44	
Major hepatic resection						
≥2 section	21	NA	94	75	75	0.088
<2 section	71	2916	88	66	54	
Alcohol						
Non-abstinence	36	2534	87	57	44	0.023
Abstinence	56	3668	91	76	66	

BMI, body mass index; GGT, Gamma-glutamyl transpeptidase; ALT, alanine aminotransferase; PT, prothrombin time; MST, median survival time; ^#^tumor differentiation: well, well-differentiated; mod, moderately differentiated; poor, poorly differentiated; MPI, microscopic portal invasion.

**Table 3:** Multivariate analysis of the overall survival.

	Hazard ratio	95% CI	*P*
Non-abstinence	2.24	1.10–4.54	0.026

CI, confidence interval.

### 3.3 Time course of postoperative serum GGT level and liver fibrosis indices

Although the median GGT levels 1 month after hepatic resection were comparable between the abstinence and non-abstinence groups (74.5 U/L and 73.5 U/L, respectively, p = 0.91), those of the non-abstinence group were significantly higher than of the abstinence group (77 U/L and 51 U/L, respectively, p = 0.031) at 24 months postoperatively. The median FIB-4 index and APRI 1 month postoperatively in the abstinence and non-abstinence groups were 2.9 and 2.6 (p = 0.84) and 0.70 and 0.58 (p = 0.28), and at 24 months postoperatively were 2.8 and 3.5 (p = 0.43) and 0.65 and 0.70 (p = 0.33), respectively (Table 4).

**Table 4:** Time course of postoperative serum GGT level, FIB-4 index, and APRI.

	Non-abstinence group (n = 36)	Abstinence group (n = 56)	*P*
GGT (U/L)			
1 month	73.5 (16–544) (n = 30)	74.5 (22–527) (n = 42)	0.91
24 months	77 (14–742) (n = 23)	51 (10–302) (n = 27)	0.031
FIB-4 index			
1 month	2.61 (0.87–8.18) (n = 34)	2.86 (0.68–7.13) (n = 52)	0.84
24 months	3.50 (1.71–13.62) (n = 30)	2.81 (0.74–13.83) (n = 37)	0.43
APRI			
1 month	0.58 (0.18–2.68) (n = 34)	0.70 (0.15–1.99) (n = 52)	0.28
24 months	0.70 (0.24–4.04) (n = 30)	0.65 (0.12–4.33) (n = 37)	0.33

GGT, gamma-glutamyl transpeptidase; FIB-4, fibrosis index based on the four factors; APRI, aspartate aminotransferase-to-platelet ratio index.

Data are presented as median values with ranges.

### 3.4 Cause of death

Overall death was confirmed in 19 patients (52.8%) in the non-abstinence group and 13 (23.2%) in the abstinence group (p = 0.0066). Incidence rates of liver-related death including HCC-specific death, liver failure, and renal failure in cirrhosis (hepatorenal syndrome) between the non-abstinence and abstinence groups were 41.7% vs. 19.6% (p = 0.032). Incidence rates of other causes of death between the two groups were 11.1% vs. 3.6% (p = 0.21) (Table 5). Details of other causes of death are shown in Supplementary Table 1.

**Table 5:** Incidence rates of death.

	Non-abstinence group (n = 36) (%)	Abstinence group (n = 56) (%)	*P*
Overall death	19 (52.8)	13 (23.2)	0.0066
Liver-related death	15 (41.7)	11 (19.6)	0.032
HCC-specific death	10 (27.8)	8 (14.3)	0.18
Liver failure	4 (11.1)	3 (5.4)	0.43
Renal failure in cirrhosis	1 (2.8)	0 (0)	0.39
Other causes	4 (11.1)	2 (3.6)	0.21

HCC, hepatocellular carcinoma.

### 3.5 Site of and treatment for first HCC recurrence

The distribution of intra- and extrahepatic recurrence and treatment for intrahepatic recurrence in the non-abstinence and abstinence groups are shown in Table 6. Intrahepatic recurrence of HCC was observed in 17 patients (47.2%) in the non-abstinence and 27 patients (48.2%) in the abstinence group (p > 0.99). Worsening of Child–Pugh grade was confirmed in 6 patients (33.3%) in the non-abstinence group and 2 patients (6.5%) in the abstinence group (p = 0.039). The proportion of treatment for intrahepatic recurrence was as follows: hepatic resection (n = 1; 5.9%), radiofrequency ablation (RFA) (n = 6; 35.3%), and transcatheter arterial chemoembolization (TACE) (n = 10; 58.8%) in the non-abstinence group, and hepatic resection (n = 5; 18.5%), RFA (n = 5; 18.5%), TACE (n = 15; 55.6%), and others (n = 2; 7.4%) in the abstinence group (p = 0.37).

**Table 6:** Site of recurrence, worsening of Child–Pugh grade at recurrence, and treatment for intrahepatic recurrence.

	Non-abstinence group (n = 36) (%)	Abstinence group (n = 56) (%)	*P*
Site of recurrence			
Overall	18 (50.0)	31 (55.4)	0.64
Intrahepatic	17 (47.2)	27 (48.2)	
Extrahepatic	1 (2.8)	4 (7.1)	
Child–Pugh grade progression(from A to B)	6 (33.3)	2 (6.5)	0.039
Treatment for intrahepatic recurrence			
Hepatic resection	1 (5.9)	5 (18.5)	0.37
RFA	6 (35.3)	5 (18.5)	
TACE	10 (58.8)	15 (55.6)	
Others	0 (0)	2 (7.4)	

RFA, radiofrequency ablation; TACE, transcatheter arterial chemoembolization.

## 4 Discussion

Multivariate analysis revealed that non-abstinence was an independent prognostic factor for OS with an approximately 2.2-fold increased risk, although RFS showed no significant difference between patients with and without alcohol abstinence. Worsening of Child–Pugh grade at intrahepatic recurrence was less frequently observed in the abstinence group. Hepatic resection tended to be more frequently performed for the treatment of HCC recurrence in the abstinence than that in the non-abstinence group. The incidence rate of liver-related death including HCC-specific death, liver failure, and renal failure in cirrhosis (hepatorenal syndrome) was significantly lower in the abstinence than that in the non-abstinence group. To the best of our knowledge, this is the first report clarifying the impact of alcohol abstinence on survival outcome after hepatic resection in patients with ALD-HCC.

Chronic alcohol consumption promotes hepatocyte injury and liver inflammation leading to the progression of liver fibrosis and hepatocarcinogenesis [11,12]. In patients with ALD, previous reports demonstrated that alcohol abstinence would reduce the increased serum levels of liver fibrosis markers and provide survival benefit of liver function preservation [26,27]. In this study, alcohol abstinence improved the OS after hepatic resection of ALD-HCC. Focusing on the cause of death, patients without abstinence showed the twofold increased risk for liver-related death compared with those with abstinence. At the time of recurrence, patients in the non-abstinence group experienced more frequent deterioration of liver function with worsening Child–Pugh grade and were less likely to receive hepatic resection for intrahepatic recurrence. Hepatic resection for HCC recurrence was reportedly associated with long-term survival [28]. These indicated that alcohol abstinence would prevent the worsening of liver function leading to not only reduced risk of liver failure progression but also increased chance of re-hepatic resection for HCC recurrence, resulting in improved prognosis postoperatively. Meanwhile, liver fibrosis indices including the FIB-4 index and APRI showed no significant differences postoperatively between patients with and without abstinence. As previously described, serum fibrosis markers were initially designed for patients with hepatitis C, and the accuracy as an index to evaluate the degree of fibrosis would differ in each serum fibrosis markers [21,26]. Clinical practice guidelines for the ALD management by the European Association for the study of the liver indicated that APRI would be of limited use in diagnosing fibrosis in patients with ALD [29]. Conversely, in this study, the serum GGT level at 24 months postoperatively was lower in the abstinence than that in the non-abstinence group. Previous reports demonstrated that the serum GGT level is the most frequently used markers to detect previous alcohol consumption, which is closely correlated with liver conditions and most useful for the ALD diagnosis [29,30]. Therefore, the serum GGT level might be useful as a biological marker of liver condition after hepatic resection in patients with ALD-HCC.

Excessive alcohol consumption would also be associated with increased risk for chronic disease progression including cardiovascular disease, cerebrovascular disease, metabolic and endocrine disease, and cancer incidence [31,32]. In this study, patients in the non-abstinence group tended to have threefold increased incidence rate of other causes of death compared with the abstinence group, such as respiratory failure, myocardial infarction, and aortic dissection. Therefore, reducing the risk for these chronic diseases induced by alcohol abstinence might contribute to improve OS.

Previous reports showed several risk factors predicting poor OS after hepatic resection of HCC, such as tumor size, vascular invasion, and tumor multiplicity as tumor-related factors, and liver cirrhosis and background liver function as liver-related factors [[33], [34], [35]]. In this study, no other tumor- or liver-related factors were identified as prognostic factors for OS. A possible reason for this is the small number of overall death. As previously described, patients with HCC who were negative for HBs Ag and anti-HCV antibody had preserved background liver function and lower frequency of liver cirrhosis [36] and showed lower risk for overall death than those of patients with hepatitis B virus- and hepatitis C virus-related HCC [3]. Our previous report also indicated the lower risk for overall death in patients negative for HBs Ag and anti-HCV antibody than patients with HCV-related HCC [37]. In this study, >90% of patients in both non-abstinence and abstinence groups had a favorable preoperative liver function with the proportion of Child–Pugh grade A, and only 32 patients died during the study period. The low incidence rate of overall death might have obscured the prognostic impact of tumor- or liver-related factors. Therefore, further accumulation of patients will reveal other prognostic factors such as tumor- or liver-related factors for survival in patients with ALD-HCC.

In terms of HCC recurrence, alcohol abstinence did not affect the RFS rate in this study. Alcohol intake has been definitely known to be involved with hepatocarcinogenesis through the liver fibrosis progression [38]. Alcohol abstinence would be expected to reduce the hepatocarcinogenic potential in patients with ALD. However, a previous report demonstrated that former drinkers who stopped 1–10 years previously still had increased risk for HCC development, whereas >10 years duration of abstinence achieved significant reduction of HCC development in patients with ALD [38]. In the present study, the median follow-up time after surgery was 45.5 months. Therefore, longer follow-up duration might clarify the favorable impact of alcohol abstinence on the risk of HCC recurrence.

In this study, proportions of >3-cm tumor size and major hepatic resection were higher in the abstinence than that in the non-abstinence group. Although all patients were advised to abstain from alcohol by primary doctor, patients with small tumor size and minor hepatic resection were less likely to achieve alcohol abstinence even after the experience of hepatic resection for HCC. Generally, lifestyle improvements, including those related to alcohol-consumption habit, require both catalysts and a strong will. Receiving a diagnosis of a large tumor and undergoing major hepatic resection would more strongly change the willingness for abstinence compared with receiving a diagnosis of a small tumor and undergoing minor hepatic resection. The change of consciousness in patients with large tumors and in those undergoing major hepatic resection might have led to the higher rate of abstinence. Therefore, the beneficial information in better survival provided by alcohol abstinence in this study may help motivate patients with ALD-HCC to abstain from alcohol after hepatic resection.

This study had several limitations that should be considered. First, because this was a retrospective study and performed in a single center, potential bias in patient enrollment may exist. Second, the total number of patients was relatively small in both groups. Third, approximately 30% of all patients dropped out during the follow-up period in the present study. However, the median follow-up periods were comparable between the patients who dropped out and those who did not drop out; therefore, the influence of patient drop-out during follow-up on long-term outcomes might be limited in the present study. Fourth, this study did not evaluate the impact of other lifestyle-related factors on the prognosis, such as body weight changes, diabetes mellitus control, and smoking status postoperatively. However, current results were based on rather long-term follow-up after hepatic resection. Thus, results of the present analysis could provide important information for the treatment of ALD-HCC. Fifth, data on alcohol consumption were retrieved from patients’ self-report or information obtained from family of patients. Such information can be biased because the true amount of alcohol consumption is often concealed. However, despite these limitations, a beneficial effect of abstinence during follow-up could be confirmed in this study.

In conclusion, alcohol abstinence might improve the long-term survival in patients with ALD-HCC undergoing hepatic resection. The current result would be important information for the management of patients with ALD-HCC.

## Ethical approval

This study was approved by the institutional ethics committee of Osaka City University Graduate School of Medicine (no. 3815).

## Consent

We gain fully informed written consent and get our institutional ethics committee approval (no. 3815).We were documented it in the paper.

## Author contribution

Daisuke Shirai and Hiroji Shinkawa made the conception and design of this study. Authors other than Daisuke Shirai contributed to the collection, analysis, and interpretation of the data. Daisuke Shirai wrote the draft manuscript, and other authors performed the critical revision of the manuscript. All authors gave final approval of the version to be published. Daisuke Shirai has overall responsibility and guarantees the scientific integrity.

## Guarantor

Daisuke Shirai and Hiroji Shinkawa have overall responsibility and guarantees the scientific integrity.

## Funding

This work was supported by Health, Labour and Welfare Policy Research Grants from the 10.13039/100009647Ministry of Health, Labour, and Welfare of Japan (Policy Research for Hepatitis Measures [H30-Kansei-Shitei-003]).

## Research registration

This study was registered with Research Registry (researchregistry6972, https://www.researchregistry.com/register-now#user-researchregistry/registerresearchdetails/60f16e12cb06e0001e5b5a9b/).

## Provenance and peer review

Not commissioned, externally peer-reviewed.

## Declaration of competing interest

All authors declare that they have no conflicts of interest.
