# Supplementary material for: Impact of alcohol abstinence on survival after hepatic resection for hepatocellular carcinoma in patients with alcohol-related liver disease
Source: Ann Med Surg (Lond). 2021 Jul 29;68:102644. doi: 10.1016/j.amsu.2021.102644 (PMC8346358; doi:10.1016/j.amsu.2021.102644)
Supplement: Multimedia component 1 [file mmc1.docx]

Supplementary table 1. Details of other causes of death

|  | Non-abstinence group  (n = 4) | Abstinence group  (n = 2) |
| --- | --- | --- |
| Respiratory failure | 1 | - |
| Myocardial infarction | 1 | - |
| Aortic dissection | 1 | - |
| Hemorrhagic duodenal ulcer | - | 1 |
| Unknown | 1 | 1 |
